# Comparing the *RETeval*^*®*^ portable ERG device with more traditional tabletop ERG systems in normal subjects and selected retinopathies

**DOI:** 10.1007/s10633-022-09903-w

**Published:** 2022-10-23

**Authors:** Jia Yue You, Allison L. Dorfman, Mathieu Gauvin, Dylan Vatcher, Robert C. Polomeno, John M. Little, Pierre Lachapelle

**Affiliations:** Department of Ophthalmology, Research Institute of the McGill University Health Center and the Montreal Children’s Hospital, Glen Site, Block E, Room EM03238 (Program Mail Drop Point EM3211), 1001 Decarie Boulevard, Montréal, QC H4A 3J1 Canada

**Keywords:** Electroretinography, *RETeval*^*®*^, Handheld stimulator, Tabletop stimulator, Recording system, Retinopathy

## Abstract

**Purpose:**

Our study aimed to determine if ISCEV standard-like ERGs recorded with the LKC *RETeval*^*®*^ portable ERG unit compared to those obtained using the more traditional tabletop unit.

**Methods:**

ERGs recorded from normal subjects and patients affected with retinal ON and OFF pathway anomalies were compared. Analysis included peak time and amplitude measurements as well as time–frequency domain analysis with the discrete wavelet transform of waveforms obtained with the two systems.

**Results:**

Although both systems were similarly able to record reliable and highly reproducible ERG responses, there were major discrepancies in ERG responses between the portable and tabletop units, pointing toward a weaker stimulation of the retinal OFF pathway with the portable *RETeval*^*®*^ unit.

**Conclusion:**

The portable *RETeval*^*®*^ unit appears to be able to record highly reproducible and diagnostically useful clinical ERGs, albeit with some significant differences in waveform composition compared to those obtained with more standard tabletop systems. Given the unknown origin of these waveform discrepancies, if left uncorrected, these differences could potentially lead to erroneous interpretation when used in the clinical context and/or compared to ERGs recorded using more traditional table top units. Clearly, more research is warranted before handheld devices, such as the *RETeval*^*®*^, can be homologated as a diagnostically sound ERG devices.

## Introduction

The flash electroretinogram (fERG) is a widely used method to assess the functional integrity of the retina in a wide variety of retinal disorders. [1, 2] Accessibility to a recording center and/or recording unit can often be a limitation in carrying out testing on all patients who could benefit from it. Recently, a portable full-field flash ERG unit has been developed (*RETeval*^*®*^, LKC Technologies, Inc., Gaithersburg, MD, USA). Initially, this unit was marketed with emphasis on its usefulness in rapidly detecting diabetic retinopathy using a 30 Hz flicker protocol [3–11]. Nowadays, the *RETeval*^*®*^ is also used to evaluate the retinal function of adult and pediatric patients using procedures claimed to be fully ISCEV-compliant by the company (https://lkc.com/products/reteval/) [12–16]. In the few studies that compared the ERG waveforms obtained with the *RETeval*^*®*^ with those obtained using a more standard system, the emphasis was mostly on amplitude and peak time comparisons. Of interest, while the timing was minimally affected, the amplitude of the responses was usually smallest with the *RETeval*^*®*^, the ratio (*RETeval*^*®*^/standard ERG system) varying roughly between 50 and 85%. Of further interest, rod responses were usually significantly more attenuated than cone responses [16], a finding also previously reported in a study [17] that compared a tabletop system (LKC UTAS-3000) with the LKC MGS-2 system (also a handheld mini-ganzfeld ERG device no longer manufactured by LKC). No studies to date looked at the *RETeval*^*®*^ responses using a time–frequency approach claimed to be superior in dissecting the ERG waveform into its primary constituents [18]. To do so, experiments were conducted in normal subjects as well as in patients affected with congenital stationary night blindness (CSNB), a retinal ON pathway anomaly [19–29], and congenital postreceptoral cone pathway anomaly (CPCPA), a retinal OFF pathway anomaly [30–32]. The results from this study were partly presented at the LIV^th^ ISCEV Symposium in Singapore [33].

## Material and methods

Informed consent was obtained from all subjects who participated in this study using forms approved by the Institutional Review Board of the McGill University Health Centre. All experiments were conducted in conformity with the Declaration of Helsinki. Prior to ERG testing, subjects underwent a complete ophthalmological examination. According to a previously described method [27, 34, 35], ERGs (OS only) were recorded from normal healthy subjects (aged 18–26, *n* = 9) as well as patients affected with congenital postreceptoral cone pathway anomaly (CPCPA; *n* = 2; aged 21 and 32 years old) and subjects with congenital stationary night blindness (CSNB; *n* = 3; aged 21, 24 and 26 years old). Briefly, eyes were dilated with tropicamide 1% solution (Mydriacyl 1%; Alcon, Fort Worth, TX, USA) and the ERGs were recorded with the active DTL fiber electrodes (27/7; X-Static silver-coated conductive nylon yarn, Sauquoit Industries, Inc., Scranton, PA, USA) positioned deep in the conjunctival sac, along with ground and reference electrodes (Grass Cup Electrodes; Natus Neurology Incorporated, Middleton, WI, USA filled with Ten/20 conductive electrode paste) pasted on the forehead and external canthi, respectively.

The ERGs recorded using the *RETeval*^*®*^ (LKC Technologies, Inc., Gaithersburg, MD, USA) were compared to those obtained using two “gold standard” tabletop units, namely: the *Espion* Profile Ganzfeld ERG (Diagnosys LLC, Lowell, MA, USA) and the LKC *UTAS-E-3000* (LKC Technologies, Inc., Gaithersburg, MD, USA). The same protocol was used with all three systems. To do so, with the assistance of the LKC support staff, the *RETeval*^*®*^ software was modified to allow the recording of photopic and scotopic ERGs of ISCEV standard quality. Photopic ERGs (*Espion* and *UTAS-3000*: interstimulus interval of 1 s, and average of 10 flashes; *RETeval*^*®*^: interstimulus interval of 1 s, and average of 10 flashes) were evoked to ganzfeld flashes of white light ranging in strength from 0.15 to 20 cd·s·m^−2^ delivered against a rod-desensitizing white light background of 30 cd·m^−2^ [34]. Scotopic recordings (20 min of dark-adaptation) were evoked to flashes of white light of 0.005, 1 and 5 cd·s·m^−2^ (Espion: interstimulus interval of 10 s, and average of 10 flashes; *RETeval*^*®*^: interstimulus interval of 10 s, and average of 10 flashes). When required, during the dark adaptation phase, electrode manipulations were performed under a dim red-light illumination. Responses contaminated with artefacts were either rejected by the experimenter at the time of testing (*Espion* recordings) or discarded automatically by the system’s software (*RETeval*^*®*^ recordings). Finally, all recordings were obtained on the same day, and the Espion- *RETeval*^*®*^ order sequence was randomized for each subject.

### Data analysis

Data analysis was limited to the a- and b-waves of the ERG which were measured from baseline to trough and from a-wave trough to peak, respectively. A paired t-test with Bonferroni correction was performed for amplitudes and peak times at each stimulus level to identify significant differences between the two systems (*p* < 0.05). Previous studies carried out in our laboratory using the discrete wavelet transform analysis (DWT) revealed that time–frequency domain analysis of the ERG can quantify morphological differences between ERG waveforms that are unnoticeable if the analysis is solely limited to amplitude and time descriptors of the ERG waveform [32, 36]. Hence, this technique was used to quantify morphological differences between the fERGs waveforms generated from the different recordings as previously published [32, 36, 37] with special attention to the ratios of two local wavelet maxima (LWM) descriptors (40b-to-20b ratios) shown to reflect the ON and OFF pathways [36]. Where applicable, measurements are reported as mean ± 1 S.D.

## Results

### Photopic ERGs

**Fig. 1:**
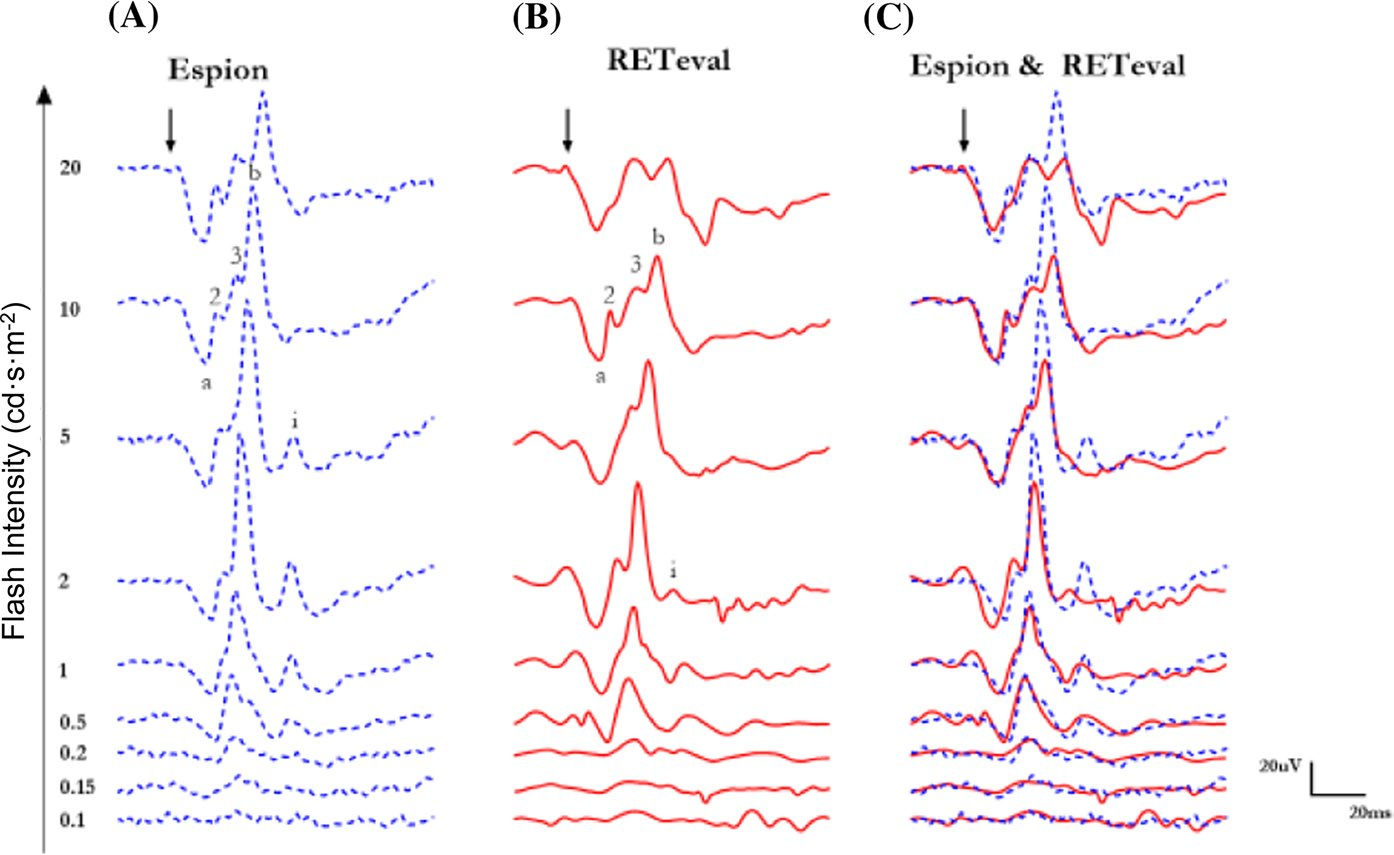
Representative photopic luminance (indicated in cd·s·m^−2^ at the left of each tracing) response function curves obtained from a normal subject recorded with the Espion (Column **A** and blue tracings) and *RETeval*^*®*^ (column **B** and red tracings) units. Both tracings are superimposed in Column **C**. ERG components identified are: a-wave (a), b-wave (b), i-wave (i) and oscillatory potentials (identified 2,3) on the rising phase of the b-wave (2,3). Vertical arrow indicates flash onset. Calibration: vertical in µvolts; horizontal in milliseconds

**Fig. 2:**
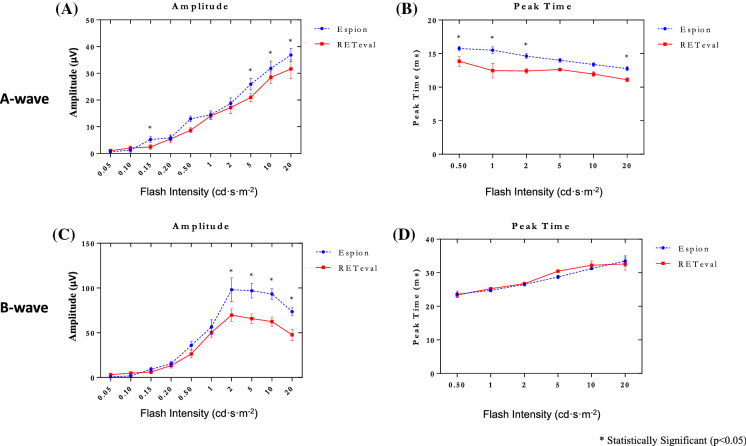
Group data for the amplitude (in µvolts: **A**,**C**) and peak time (in milliseconds: **B**,**D**) measurements of the photopic a-wave (**A**,**B**) and b-wave (**C**,**D**) recorded to progressively brighter stimuli (abscissa in cd·s·m^−2^) with the *RETeval*^*®*^ (red lines) and *Espion* (blue lines) systems. Amplitude and peak times are shown for the a-wave (panels **A** and **B**, respectively) and for the b-wave (panels **C** and **D**, respectively). Asterisks identify statistically significant differences between the *RETeval*^*®*^ and *Espion* measurements. Note that with both systems, the maximal b-wave amplitude (i.e., peak of the Photopic Hill) is reached for the same flash stimulus (i.e., 2 cd·s·m.^−2^)

Representative photopic ERG waveforms recorded from control subjects with the *Espion* (A) and *RETeval*^*®*^ (B) units are shown separately at Fig. 1A and 1B and superimposed at Fig. 1C, respectively. With both systems, the use of progressively brighter flashes elicits the expected growth followed by a decay in b-wave amplitude (Fig. 2C), a phenomenon known as the Photopic Hill [27, 30, 34, 35, 38, 39]. However, depending on the strength of the flash used, the morphologies of the resulting Espion and *RETeval*^®^ waveforms can be strikingly different (Fig. 1C). More specifically, the overlay of responses illustrated in Fig. 1C reveals that while ERG waveforms are perfectly superimposable at lower stimulus levels (up to 1 cd·s·m^−2^), there are strikingly different in amplitudes, peak times and morphologies for flash stimuli of 2 cd·s·m^−2^ and above. Of note, these morphological differences are most noticeable on the descending phases of the b-waves (i.e., from the b-wave peak down to the baseline) as seen at Fig. 2C, while the ascending phases of the b-waves (i.e., from the a-wave trough to the peak of the b-wave) are nearly identical. Also, while the *Espion* ERGs show a large amplitude i-wave in responses evoked to the 1–5 cd·s·m^−2^ stimuli, in the *RETeval*^*®*^ ERGs this post-b-wave component is minimal to nonexistent. The amplitude and timing differences are best illustrated at Fig. 2 where group data are reported. Figure 2A reveals that while the amplitudes of the *Espion* and *RETeval*^*®*^ a-waves are not significantly different from each other for flashes between 0.05 and 2 cd·s·m^−2^ (except for 0.15 cd·s·m^−2^), they are different for ERGs evoked to stronger stimuli (i.e., 5, 10 and 20 cd·s·m^−2^; *p* < 0.05). As shown at Fig. 2B, peak time differences also distinguish the *Espion* and *RETeval*^*®*^ a-waves, where the *Espion* a-wave is significantly (*p* < 0.05) delayed compared to *RETeval*^*®*^ for all stimuli except 5 and 10 cd·s·m^−2^, a feature that is also well illustrated with the tracing superposition shown at Fig. 1C. As alluded to above, both systems generate a Photopic Hill-like b-wave luminance response as illustrated at Fig. 2C. The results show that there are no significant differences in the *Espion* and *RETeval*^*®*^ Photopic Hills for b-wave amplitudes making the raising phase of the Photopic Hill (i.e., between 0.05 and 1 cd·s·m^−2^). However, for stronger flashes (i.e., peak and falling phase of the Photopic Hill), the *Espion* b-waves are always significantly larger than that of the *RETeval*^*®*^*,* the largest amplitude difference being measured in ERGs evoked to the 5 cd·s·m^−2^ (*Espion:* 96.88 ± 22.7µvolts; *RETeval*^*®*^: 65.86 ± 14.8µvolts; *P* < 0.05). Of note, the peak of the photopic Hill (Fig. 2C) is reached for the same flash stimulus (i.e., 2 cd·s·m^−2^) with both system and in both cases it is then followed by a gradual reduction in b-wave amplitude as one would expect with the Photopic Hill phenomenon. In contrast, as shown at Fig. 2D, the peak times of the *Espion* and *RETeval*^*®*^ b-waves are not significantly (*P* > 0.05) different from each other.

### Scotopic ERGs

**Fig. 3:**
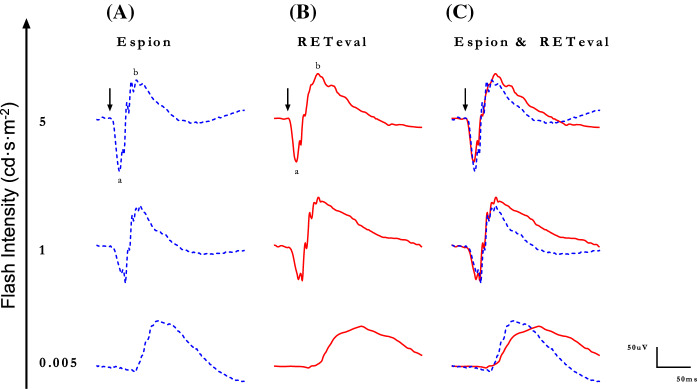
Representative scotopic luminance (indicated in cd·s·m^−2^ at the left of each tracing) response function curves obtained from a normal subject recorded with the Espion (Column **A** and blue tracings) and *RETeval*^*®*^ (column **B** and red tracings) units. Both tracings are superimposed in Column **C**. ERG components identified are: a-wave (a) and b-wave (b). Vertical arrow indicates flash onset. Calibration: vertical in µvolts; horizontal in milliseconds

Representative scotopic [rod-mediated (0.005 cd·s·m^−2^) and rod-cone-mediated (1 and 5 cd·s·m^−2^)] ERG waveforms recorded from control subjects with the *Espion* (A) and *RETeval*^*®*^ (B) units are shown separately in Fig. 3A and B and superimposed in Fig. 3C, respectively. While the rod-mediated responses do show significant timing differences, the morphologies of the mixed rod-cone mediated ERGs are more similar, at least from flash onset to b-wave peak, while the descending phase of the b-wave do show some differences similar to what is reported above for the photopic ERGs.

**Fig. 4:**
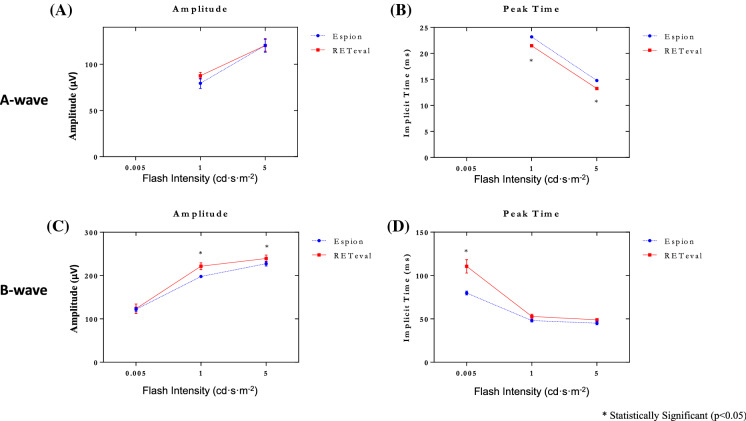
Group data for the amplitude (in µvolts: **A**,**C**) and peak time (in milliseconds: **B**,**D**) measurements of the scotopic a-wave (**A**,**B**) and b-wave (**C**,**D**) recorded to progressively brighter stimuli (abscissa in cd·s·m^−2^) with the *RETeval*^*®*^ (red lines) and *Espion* (blue lines) systems. Note that no reproducible a-wave could be measured in response to the dimmest flash (0.005 cd·s·m^−2^). Amplitude and peak times are shown for the a-wave (panels **A** and **B**, respectively) and for the b-wave (panels **C** and **D**, respectively). Asterisks identify statistically significant differences between the *RETeval*^*®*^ and *Espion* measurements

The amplitude and timing differences are best illustrated in Fig. 4 where group data is reported. Figure 4A and B reveals that while the amplitudes of the *Espion* and *RETeval*^*®*^ scotopic a-waves are indistinguishable from each other (Fig. 4A), the peak times of the *RETeval*^*®*^ a-waves are significantly faster than those of the *Espion* (Fig. 4B). The latter contrasts with b-wave measurements where the amplitudes of the *Espion* and *RETeval*^*®*^ b-waves (Fig. 4C) are not significantly different in response to the pure rod stimulus (i.e., 0.005 cd·s·m^−2^) while the amplitude of the *Espion* mix rod-cone b-waves (1 and 5 cd·s·m^−2^) are significantly smaller than those obtained with the *RETeval*^*®*^ system [(*p* < 0.05), largest difference at 1 cd·s·m^−2^, mean *Espion* amplitude = 197.8 ± 5.3 µvolts, mean *RETeval*^*®*^ amplitude = 221.6 ± 17.1 µvolts]. The latter contrast with peak time measurements (Fig. 4D) where the timing of the *Espion* and *RETeval*^*®*^ b-waves is not significantly different from each other in responses evoked to the 1 and 5 cd·s·m^−2^ while the timing of the *Espion* b-wave is significantly faster than that of the *RETeval*^*®*^ in response to the rod-mediated stimulus (i.e., 0.005 cd·s·m^−2^).

### Analysis of the photopic ERG in the time–frequency domain

**Fig. 5:**
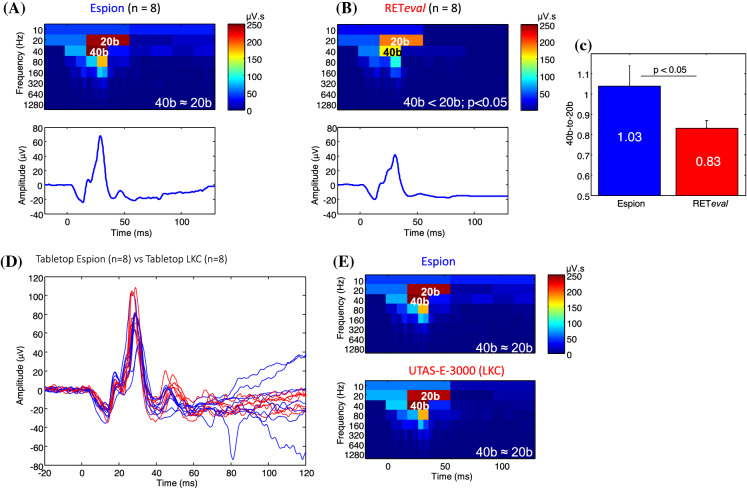
Scalograms of the discrete wavelet transform (DWT) obtained from ERG waveforms at 5 cd·s·m^−2^ recorded from normal subjects using the *Espion* (**A**) and *RETeval*^*®*^ (**B**) units. (**C**) The *RETeval*^*®*^ 40b:20b (or OFF:ON) ratio is significantly smaller than that measured in ERGs recorded with the *Espion*. (Data presented as mean ± 1 S.D.) Comparison of normal ERG responses recorded using two tabletop systems (all subjects went through the same procedure with the two systems), namely: the *Espion* (blue tracings) and LKC *UTAS-3000* (red tracings) units. (**E**) Scalograms of DWT for ERGs recorded with the *Espion* (top) and LKC *UTAS-3000* (bottom) units. No significant differences were found between the two tabletop units, both systems yielding identical 40b:20b ratios. Color calibration of scalograms in µV·s

Time–frequency scalograms [generated from the discrete wavelet transform (DWT) analysis] of normal photopic ERGs [evoked at the peak of the Photopic Hill (i.e., 5 cd·s·m^−2^ stimulus)] obtained with the *Espion* and *RETeval*^*®*^ systems are compared at Fig. 5A and B, respectively. The 20b and 40b descriptors (previously shown to be associated to the ON and OFF retinal pathways, respectively [30, 35]), are those showing largest energy difference between the two systems. While the *Espion* 40b:20b ratio is, as previously reported [32, 36], close to unity at 1.03 ± 0.09, the *RETeval*^*®*^ 40b:20b ratio is significantly (*p* < 0.05) decreased to 0.83 ± 0.04, due to the reduced contribution of the 40b (OFF) descriptor, a finding that suggests a weaker stimulation of the retinal OFF pathway with the *RETeval*^*®*^ system. The latter claim is also supported with results shown at Fig. 5D and E showing a comparison between two tabletop units (i.e., LKC *UTAS-E-3000* and *Espion*). Both systems evoked photopic ERGs of similar morphologies (Fig. 5D) and equivalent frequency domain composition notably the 20b and 40b descriptors, resulting in nearly identical 40b:20b ratios (*Espion*: 1.03 ± 0.09 and LKC *UTAS-E-3000*: 1.05 ± 0.06; *p* > 0.05), as illustrated at Fig. 5E.

### Comparing the photopic ERGs in selected retinopathies

**Fig. 6:**
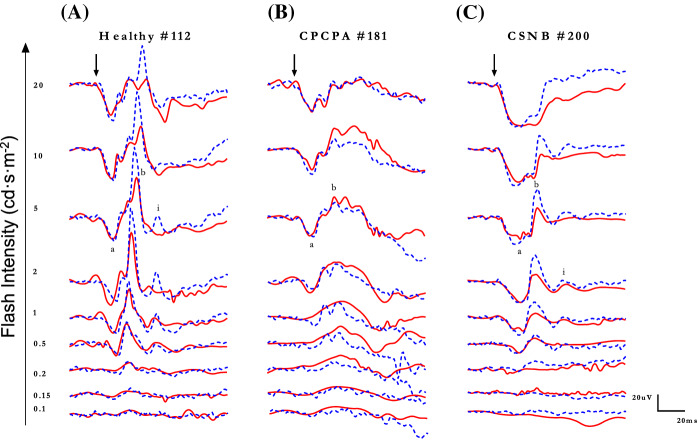
Representative photopic luminance (indicated in cd·s·m^−2^ at the left of each tracing) response function curves obtained from a normal subject Column **A**) and patients affected with CPCPA (Column **B**) and CSNB (Column **C**) recorded with the Espion (blue dotted tracings) and *RETeval*^*®*^(red solid tracings) units (both tracings are superimposed). ERG components identified are: a-wave (a), b-wave (b) and i-wave (i). Vertical arrow indicates flash onset. Calibration: vertical in µvolts; horizontal in milliseconds

**Fig. 7:**
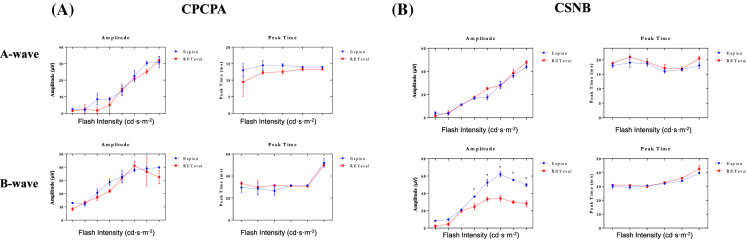
Group data for the amplitude (in µvolts) and peak time (in milliseconds) measurements of the photopic a-wave and b-wave of CPCPA (**A**) and CSNB (**B**) patients recorded to progressively brighter stimuli (abscissa in cd·s·m^−2^) with the *RETeval*^*®*^ (red lines) and *Espion* (blue lines) systems. Asterisks identify statistically significant differences between the *RETeval*^*®*^ and *Espion* measurements

In light of the above findings suggesting that, unlike the gold standard tabletop ERG units, the handheld *RETeval*^*®*^ system did not appear to stimulate the ON and OFF retinal pathways equally (i.e., 40b:20b ratio < 1.0) in normal subjects, we compared the photopic ERGs obtained from patients known to have an ON or OFF retinal pathway anomaly, namely congenital stationary night blindness (CSNB) and congenital postreceptoral cone pathway anomaly (CPCPA), respectively. Representative photopic ERGs (*Espion* and *RETeval*^*®*^ tracings are superimposed) evoked to progressively brighter stimuli from a normal subject and patients affected with CPCPA or CSNB are illustrated at Fig. 6A, B, and C, respectively. Compared to the normal tracings (Fig. 6A), those obtained from the CPCPA patient (Fig. 6B) show less amplitude and morphology discrepancies between the *Espion* and *RETeval*^*®*^ responses, especially in responses evoked to the brighter stimuli (i.e., 2–20 cd·s·m^−2^). In contrast, in most tracings shown, significant amplitude differences are observed between *Espion* and *RETeval*^*®*^ responses recorded from the CSNB patient (Fig. 6C). This is best exemplified at Fig. 7 where the group data is presented. In CPCPA, the *Espion* and *RETeval*^*®*^ a- and b-wave amplitudes and peak times are indistinguishable from each other, irrespective of the strength of the flash stimulus (Fig. 7A; *p* > 0.05). In contrast, while in CSNB the *Espion* and *RETeval*^*®*^ ERGs have identical a-waves (amplitudes and timings) and b-wave timings, the amplitude of the *RETeval*^*®*^ b-wave is significantly reduced compared to the *Espion* b-wave, for flash stronger than 0.5 cd·s·m^−2^ (Fig. 7B); the largest amplitude difference being observed in responses evoked to the 5 cd·s·m^−2^ (*Espion*: 61.93 ± 5.2 µvolts; *RETeval*^*®*^: 34.24 ± 5.7 µvolts).

### Comparing the scotopic ERGs in selected retinopathies

**Fig. 8:**
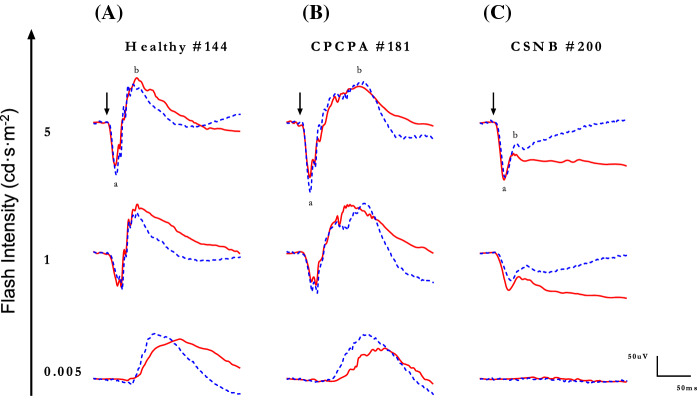
Representative scotopic luminance (indicated in cd·s·m^−2^ at the left of each tracing) response function curves obtained from a normal subject Column **A**) and patients affected with CPCPA (Column **B**) and CSNB (Column **C**) recorded with the Espion (blue dotted tracings) and *RETeval*^*®*^(red solid tracings) units (both tracings are superimposed). ERG components identified are: a-wave (a) and b-wave (b). Vertical arrow indicates flash onset. Calibration: vertical in µvolts; horizontal in milliseconds

**Fig. 9:**
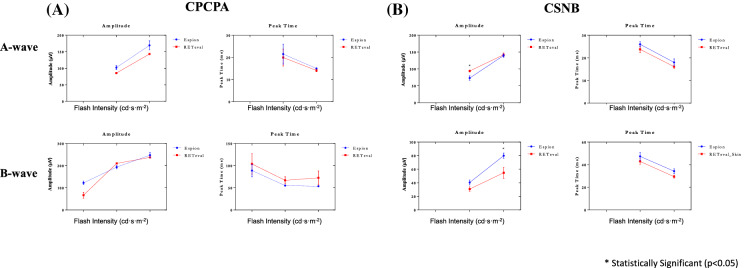
Group data for the amplitude (in µvolts) and peak time (in milliseconds) measurements of the scotopic a-wave and b-wave of CPCPA (**A**) and CSNB (**B**) patients recorded to progressively brighter stimuli (abscissa in cd·s·m^−2^) with the *RETeval*^*®*^ (red lines) and *Espion* (blue lines) systems. Note that no reproducible a-wave could be measured in response to the dimmest flash (0.005 cd·s·m^−2^). Asterisks identify statistically significant differences between the *RETeval*^*®*^ and *Espion* measurements

Representative scotopic [rod-mediated (0.005 cd·s·m^−2^) and rod-cone-mediated (1 and 5 cd·s·m^−2^)] ERG waveforms recorded with the *Espion* (A) and *RETeval*^*®*^ systems (tracings are superimposed) from a normal subject and patients affected with CPCPA and CSNB are shown at Fig. 8A, B, and C, respectively. While the rod-mediated (i.e., 0.005 cd·s·m^−2^ stimulus) *Espion* and *RETeval*^*®*^ ERGs recorded from the normal and CPCPA subjects do show significant timing differences, as expected, no response could be recorded from the CSNB patient, irrespective of the system used. Similarly, while both systems generate similar (in peak time, amplitude and wave morphology) mix rod-cone ERGs in CPCPA (Fig. 8B; tracings 1 and 5 cd·s·m^−2^), more variability is observed with the CSNB responses (Fig. 8C). These amplitude and timing differences are best illustrated at Fig. 9 where group data is reported. Again, as shown in Fig. 9A, there are no significant differences in amplitudes and peak times for the a- and b-waves of the *Espion* and *RETeval*^*®*^ scotopic ERGs recorded from CPCPA patients. Similarly, while the timing of the *Espion* and *RETeval*^*®*^ a- and b-waves are indistinguishable from each other in CSNB (Fig. 9B), the amplitudes are; the *RETeval*^*®*^ yielding the largest a-wave in response to the 1 cd·s·m^−2^ stimulus and the smallest b-wave in response to the 5 cd·s·m^−2^ (*Espion*: 80.0 ± 6.7 µvolts; *RETeval*^*®*^: 54.8 ± 14.0 µvolts; *p* < 0.05).

### Analysis of the abnormal photopic ERG in the time–frequency domain

**Fig. 10:**
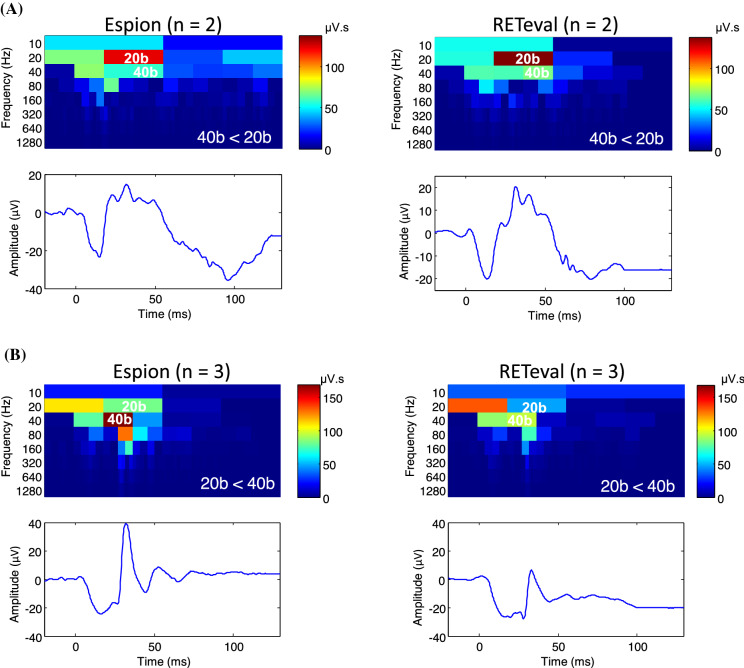
Scalograms (with accompanying ERG waveforms) of the discrete wavelet transform (DWT) obtained from ERG waveforms at 5 cd·s·m^−2^ recorded from CPCPA (**A**, *N* = 2) and a CSNB (**B**, *N* = 3) patients using the *Espion* (left column) and *RETeval*^*®*^ (right column) units. 40b:20b relationship is indicated at the bottom left of each scalogram. Color calibration of scalograms in µV·s

The discrete wavelet transform (DWT) scalograms obtained from *Espion* and *RETeval*^*®*^ ERGs evoked at the peak of the Photopic Hill (i.e., 5 cd·s·m^−2^ stimulus) in CPCPA and CSNB patients are shown at Fig. 10A and B, respectively. While in normal subjects, the 20b and 40b components equally contribute to the building of the photopic ERG (see Fig. 5), in CPCPA both systems markedly attenuated the 40b component, resulting in significantly lower than normal 40b:20b ratios (*Espion*: 0.46 ± 0.03; *RETeval*^*®*^: 0.49 ± 0.00; *p* > 0.05). In contrast, while the 20b components is the one most significantly attenuated in ERGs recorded from CSNB patients (Fig. 10B), the resulting *Espion* and *RETeval*^*®*^ 40b:20b ratio differ (*Espion*: 2.07 ± 0.24; *RETeval*^*®*^: 1.57 ± 0.19), suggesting that the expected attenuation of the 20b component was, in *RETeval*^*®*^ recordings only, also accompanied by a concomitant attenuation of the 40b component.

## Discussion

The purpose of this study was to determine if the *RETeval*^*®*^ system could be used interchangeably with the more standard tabletop unit to record ISCEV standard-like clinical ERGs. In order to achieve our goal, we compared full field photopic and scotopic flash ERGs obtained with the two systems, with the same electrodes and from the same participants, including healthy subjects as well as subjects previously diagnosed with selected retinopathies. To our knowledge this is the first time that such a comparison is attempted, since most studies to date on the *RETeval*^*®*^ system only considered the flicker ERG protocol [3–10, 14]. In fact, only two studies in the literature incorporated the use of more standard ERGs, namely: Asakawa et al. who used cone- and rod-mediated ERGs [40] and Wu et al. study on the photopic negative response (PhNR) [41]. Although in the latter study, reference is made to a previous study of the same group [42] where the PhNR responses were recorded using a tabletop system (*Espion* E2/ColorDome; Diagnosys LLC, Lowell, MA, USA), there were no direct comparisons made between the two systems (i.e., *RETeval*^*®*^ and *Espion*). Of interest, they observed that the repeatability of the handheld device was three times better than that of their tabletop system, a feature that they attributed to the larger number of sweeps composing the *RETeval*^*®*^ averages (*N* = 200 sweeps) compared to the *Espion* averages (*N* = 10 sweeps).

Our results clearly demonstrate that the *RETeval*^*®*^ system can record ISCEV standard-like ERGs of good quality and reproducibility. For example, despite the amplitude difference being the largest at the peak of the Photopic Hill (*Espion:* 96.88 ± 22.7; *RETeval*^*®*^: 65.86 ± 14.8), the coefficient of variation (i.e., CV% = Standard Deviation ÷ Mean) are identical (*RETeval*^*®*^: 22.5; *Espion*: 23.4). Similarly, despite subtle differences, the pathognomonic characteristics of the photopic ERGs of CPCPA and CSNB could also be evidenced in *RETeval*^*®*^ recordings.

However, notwithstanding the above, major differences were noted in the morphology and frequency composition of photopic ERGs as well as in the Photopic Hills recorded with the two systems, which taken together point to a weaker (or less efficient) stimulation of the OFF retinal pathway with the *RETeval*^*®*^ system. In normal subjects, the descent of the Photopic Hill of the *RETeval*^*®*^ photopic ERGs showed obvious morphological anomalies (i.e., reduced b-wave peak amplitude, slower return to baseline, absent i-wave) in a region of the photopic ERG that was previously shown to be associated to the OFF retinal pathway [37, 43–45]. Similarly, we also showed that the largest intersystem discrepancy is seen in responses evoked to strongest flash (i.e., the peak and descent of the Photopic Hill; Fig. 2C), where the *RETeval*^*®*^ ERGs are markedly reduced compared to the *Espion* ERGs. In a previous study, we showed that, while the energy level of both the ON and OFF components of the short flash ERG increased with increasingly stronger flash stimuli, a large enhancement of the OFF component (that far exceeded that measured for the ON component) was measured in ERGs evoked to the strongtest stimuli, suggesting a facilitation of the ERG OFF component with stronger flashes [37]. Our claim of a weaker stimulation of the OFF retinal pathway with the *RETeval*^*®*^ system could explain the drop in amplitude noted for the strongest flash. Finally, our demonstration that the reduced 40b:20b ratio in *RETeval*^*®*^ photopic ERG of normal subjects was due to the specific attenuation of the 40b components previously shown to be associated to the OFF retinal pathway [37] would also support our claim of a less efficient OFF retinal pathway stimulation with the *RETeval*^*®*^ unit. The latter claim is also supported with our findings from our selected patients. Comparing photopic ERGs obtained from patients affected with CSNB [a disorder of the ON pathway [19–24] (where ERGs are mostly generated by the OFF retinal pathway)] and CPCPA [a disorder of the OFF pathway [30, 31, 35] (where ERGs are mostly generated by the ON retinal pathway)], revealed that the largest intersystem differences were observed in responses recorded from CSNB patients. In contrast, we could not evidence statistically significant intersystem differences in ERGs recorded from our CPCPA patients.

It is difficult at this point to identify the reason(s) why the *RETeval*^*®*^ system cannot stimulate the OFF retinal pathway as efficiently as the tabletop units do. In that respect it is interesting to note that when the *Espion* system is compared to the tabletop LKC system (LKC *UTAS-E-3000*) both systems generate ERG tracings that are indistinguishable from each other (Fig. 5D). The latter is also confirmed with the results obtained from the time–frequency analysis which showed identical 40b:20b ratios (*Espion*: 1.03 ± 0.09 and *UTAS-E-300*: 1.05 ± 0.06; *p* > 0.05) compared to a significantly lower ratio (0.83 ± 0.04; *p* < 0.05) for the *RETeval*^*®*^. This confirmed that the unequal contribution of the 20b and 40b descriptors to the making of the *RETeval*^*®*^ photopic ERG that we evidenced in the present study, was solely limited to the handheld LKC device.

Among the factors that could explain a weaker stimulation of the OFF retinal pathway with the *RETeval*^*®*^, one also wonders if the size of the Ganzfeld could have contributed. In a previous study which compared another handheld ERG device (*Ephios*) with another tabletop system (*VERIS*), the authors concluded that the ERG waveforms generated with the handheld unit were comparable (nearly identical coefficient of variation) to those obtained with the tabletop device [46]. Of note however, only peak time and amplitude measurements of normal scotopic responses were considered, that is ERGs that we also showed to yield the highest intersystem similarity, in normal (Figs. 3 and 4) and diseased (Figs. 8 and 9) retinas. Thus, from this we cannot exclude the possibility that a smaller Ganzfeld that equips handheld devices could not have contributed to the intersystem ERG discrepancies reported herein.

In the present study, our results show that the maximal amplitude of the photopic b-wave (i.e., peak of the Photopic Hill) reached with the *Espion* (96.88 ± 22.7µvolts) is significantly larger (*P* < 0.05) than that reached with the *RETeval*^*®*^ (65.86 ± 14.8µvolts). The maximum b-wave reached with the *RETeval*^*®*^ is thus 68% of that reached with the *Espion*. Of interest, as shown at Fig. 2C, 1-Both Photopic Hill peaks are reached for the same flash strength, 2-The b-waves obtained for weaker flashes (ascending limb of the Photopic Hill) do not reveal significant differences and, 3- The *RETeval*^*®*^ b-waves obtained past the Photopic Hill peak (i.e., descending limb of the Photopic Hill) are all significantly smaller that those obtained with the *Espion* system. We believe that the latter results also point to an abnormal OFF pathway contribution to the genesis of the *RETeval*^*®*^ photopic ERG for the following reasons. Firstly, in a previous study of ours [34] we showed that at the beginning of the Light Adaptation Effect (LAE) the amplitude of the b-wave at the peak of the Photopic Hill is 65.4 ± 8.8% of that measured at control (i.e., fully light adapted subjects), while no significant amplitude differences were measured for responses evoked for weak flash (i.e., ascending limb of the Photopic Hill); thus the same pattern as above when the *RETeval*^*®*^ and *Espion* Photopic Hills are compared. In this study we also demonstrated, using a mathematical modelization of the Photopic Hill, that while the Gaussian function grew significantly with LA, the logistic growth function remained basically unchanged. A previous report showed that the Gaussian and the logistic growth functions assessed the contribution of the OFF and ON pathways, respectively [47], suggesting that the OFF pathway was significantly more affected than the ON pathway by the LAE (possibly due to a gradual release of inhibition of the ON pathway on the OFF pathway). We believe that the similarity in Photopic Hill growth and decay when the *RETeval*^*®*^ and *Espion* Photopic Hills (see Fig. 2C) are compared would also support the same conclusion that the OFF pathway is more suppressed in responses evoked with the *RETeval*^*®*^. Secondly, we have also previously shown that the maximal amplitude (Peak of the Photopic Hill) reached with a red flash (74.3 ± 20.2 uV) was significantly smaller than that reached with a white stimulus (92.2 ± 21.3 uV; *p*˂0.05) while the PH peak obtained to blue (95.1 ± 20.0 uvolts) and green (90.6 ± 22.6 uV) flashes were not significantly different from white [48]. The smaller b-waves evoked to the red flashes were due to a nearly extinguished OP_4_ [i.e., the OP that forms the last segment of the ascending limb of the suprathreshold photopic b-wave [49]] a feature also observed in our *RETeval*^*®*^ ERGs (see Fig. 1C), but never observed in responses evoked to white, blue or green flashes [48]. Of note, this last segment of the ascending limb of the suprathreshold photopic b-wave (or OP_4_) was shown to be associated to the OFF retinal pathway [37, 43, 44]. While both the *Espion* and the *UTAS-3000* make use of Xenon tubes to generate the white flashes used in this study, the *RETeval*^*®*^ uses a combination of red, green and blue LEDs to generate the white stimulus [50]. It could be that an excess of red in the mixture could explain the deficit in OFF pathway stimulation with the *RETeval*^*®*^.

Given that the precise nature and origin of these differences which, if left uncorrected, could lead to erroneous interpretation when used in the clinical context (especially when comparing *RETeval*^*®*^ results with those published that were obtained using a tabletop system), more research is warranted before handheld devices such as the *RETeval*^*®*^ can be homologated as diagnostically sound ERG devices.

## Declarations

### Statement on the welfare of animals

This article does not contain any studies with animals performed by any of the authors.
